# Molecular biology of canine parainfluenza virus V protein and its potential applications in tumor immunotherapy

**DOI:** 10.3389/fmicb.2023.1282112

**Published:** 2023-12-20

**Authors:** Huai Cheng, Hewei Zhang, Huanchang Cai, Min Liu, Shubo Wen, Jingqiang Ren

**Affiliations:** ^1^Wenzhou Key Laboratory for Virology and Immunology, Institute of Virology, Wenzhou University, Wenzhou, China; ^2^College of Food and Drugs, Luoyang Polytechnic, Luoyang, China; ^3^Animal Diseases and Public Health Engineering Research Center of Henan Province, Luoyang, China; ^4^Preventive Veterinary Laboratory, College of Animal Science and Technology, Inner Mongolia Minzu University, Tongliao, China

**Keywords:** canine parainfluenza virus, V protein, molecular mechanism, structure, viral replication, immune escape, tumor immunotherapy

## Abstract

Canine parainfluenza virus (CPIV) is a zoonotic virus that is widely distributed and is the main pathogen causing canine infectious respiratory disease (CIRD), also known as “kennel cough,” in dogs. The CPIV-V protein is the only nonstructural protein of the virus and plays an important role in multiple stages of the virus life cycle by inhibiting apoptosis, altering the host cell cycle and interfering with the interferon response. In addition, studies have shown that the V protein has potential applications in the field of immunotherapy in oncolytic virus therapy or self-amplifying RNA vaccines. In this review, the biosynthesis, structural characteristics and functions of the CPIV-V protein are reviewed with an emphasis on how it facilitates viral immune escape and its potential applications in the field of immunotherapy. Therefore, this review provides a scientific basis for research into the CPIV-V protein and its potential applications.

## 1 Introduction

Canine parainfluenza virus (CPIV) is a single-stranded negative RNA virus with non-segmented segments that belongs to the *Paramyxoviridae* family and *Rubulavirus* genus (Yang, 2022). When the virus was first isolated from rhesus monkey and cynomolgus monkey kidney cells, it was named as simian virus 5 (SV5) (Hull et al., 1956). However, because it is a canine pathogen that causes infectious tracheobronchitis and even secondary pneumonia and other diseases of the respiratory tract, resulting in ‘kennel cough’, it is also called CPIV (Yang, 2022). Notably, CPIV infection has been reported in cats, hamsters, guinea pigs, pigs and humans, but most of these infections do not lead to disease (Charoenkul et al., 2021; Ibrahim et al., 2022; Wang et al., 2023). In recent years, arthropod ticks have been mechanical carriers of CPIV (Yang et al., 2022). Since CPIV has been isolated from multiple species and because all its natural hosts have not been identified, CPIV was named parainfluenza virus 5 (PIV5) by the International Committee on Virus Classification in 2009 (Chen, 2018). In 2016, the virus was renamed mammalian orthorubulavirus 5 (Yang, 2022). Since CPIV is commonly used to represent the virus causing kennel cough in veterinary medicine, it is referred to as CPIV in this paper.

The CPIV genome is 15,246 bp long, which is the smallest paramyxovirus genome (Wang et al., 2023), and follows the “rule of six”, which means that efficient replication of most paramyxoviruses occurs only when the length of the viral genome is a multiple of six (Kolakofsky et al., 1998). From the 3′ end to the −5′ end, the CPIV genome sequentially encodes a nucleocapsid protein (NP), a phosphoprotein (P), a matrix protein (M), a small hydrophobic protein (SH), a hemagglutinin neuraminidase (HN) and a large polymerase protein (L). The V protein is a viral nonstructural protein produced by RNA editing of the P gene (Chen, 2018). Many studies have shown that the V protein not only controls viral RNA synthesis and promotes viral replication but is also extensively involved in the interaction between the virus and host, preventing apoptosis and inhibiting the interferon (IFN) response, immune-related cytokine expression, and other processes.

## 2 Molecular mechanism underlying CPIV-V protein effects

Overlapping genes, also known as overprinting genes, are defined as two or more genes that share a single DNA sequence. Due to the limits on viral genome length, overprinting is widespread in the viral genome (Wright et al., 2022), and differentiation between genes can occur in gene transcription or mRNA translation. At the transcriptional level, viruses typically employ a cotranscriptional RNA editing strategy by adding several nontemplated nucleotides into nascent RNA during the process of transcription (Rao et al., 2020). At the translation level, viruses undergo overprinting through noncanonical translation initiation and extension (Sorokin et al., 2021). The process mainly involves non-AUG codon initiation, ribosome frameshifting and other mechanisms (Douglas et al., 2021).

**Figure 1:**
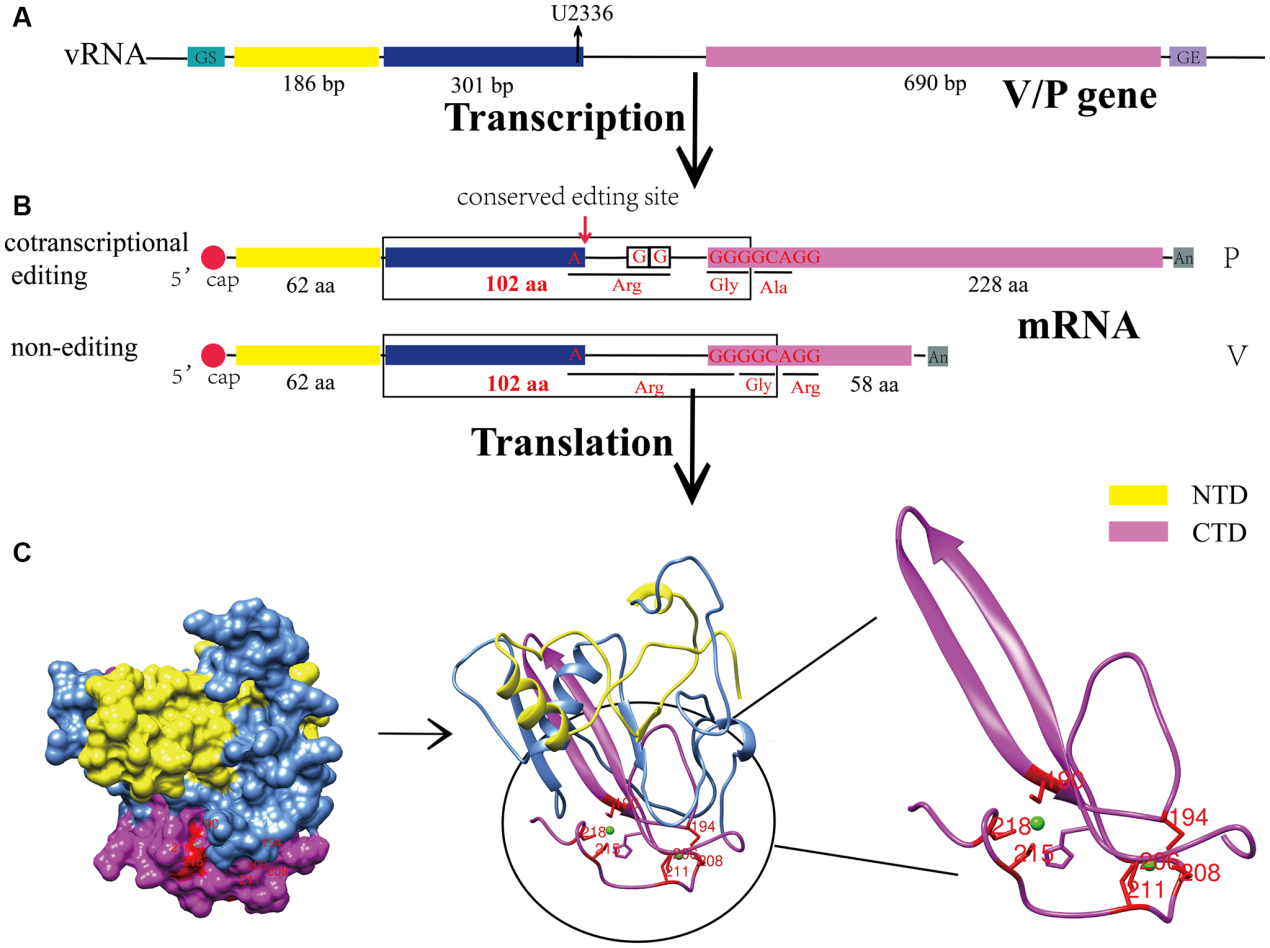
Biosynthesis process and structural characteristics of the CPIV-V protein. **(A)** Genomic characteristics of the V/P gene. U2336 is the conserved editing site in the viral genome. **(B)** V/P mRNA is produced by different modes of transcription. **(C)** Sphere model, cartoon representation and C-terminal zinc finger domain of the CPIV-V protein. All protein structures were created on UCSF Chimera. bp, base pairs; aa, amino acid; vRNA, viral RNA; GS, gene start; GE, gene end; NTD, N-terminal domain; CTD, C-terminal domain.

CPIV belongs to the paramyxovirus family, and many studies have shown that RNA editing occurs in the transcription of the paramyxovirus P gene, which produces V, W and other proteins that antagonize the host innate immune response (Kolakofsky, 2016). Currently, two models have been established for this editing process: the P and V models (Douglas et al., 2021). The former involves the P protein encoded by the original gene sequence, while V, W and other proteins are translated after the insertion of one or two guanylate acids (G) into the conserved region of the transcribed mRNA of the P gene. In the V model, the V protein is encoded by the original gene sequence, the mRNA encoding the W protein acquires a G that is inserted during transcription, and P carries two G inserted at the same location. CPIV is a V model virus. During V gene transcription, two unpaired G are added at the 551th nucleotide of its mRNA sequence, and thus, the open read frame (ORF) is changed to generate P mRNA (Figure 1B), and the number of V gene transcripts is slightly higher than that of the P gene (Thomas et al., 1988; Wang et al., 2023). Other viruses in the same genus, such as human parainfluenza virus type 2 (HPIV2) (Ohta et al., 2021), belong to the V model. However, the well-characterized Newcastle disease virus (NDV) (Jadhav et al., 2020) and measles virus (MeV) (Nagano et al., 2020) are P model viruses. Although the mechanism underlying V protein biosynthesis varies among paramyxoviruses, all V proteins share an N-terminal domain (NTD) with the P protein, and the C-terminal domain (CTD) is highly conserved among paramyxoviruses. Notably, it was originally found that the V protein is not necessary for viral replication and was naturally called an accessory protein (Peluso et al., 1977); however, in 1995, Paterson confirmed that the V protein is also a structural viral protein and a component of the nucleocapsid core through polypeptide analysis and immunoelectron microscopy (Paterson et al., 1995), revealing the potential functions of the V protein in the viral cycle.

## 3 Structure of the V protein

The V protein of CPIV contains 222 amino acids, including the NTD, which shares 164 amino acid residues with the P protein, and a unique cysteine-rich CTD (Figure 1A) (Wang et al., 2023). The CTD has a conserved motif containing seven cysteines, which can bind two zinc atoms to form a zinc finger structure (Figure 1C) (Paterson et al., 1995). Many studies have shown that the C-terminal zinc finger domain of the V protein is crucial for its normal function. Initial studies found that it can inhibit the transcription of viral RNA (Yang et al., 2015). Moreover, it can inhibit the apoptosis of host cells (Sun et al., 2004) and contributes to the regulation of viral replication (He et al., 2002). In addition, the zinc finger structure can interact with damage-specific DNA binding protein 1 (DDB1), causing a conformational change in DDB1, which enables binding to the signal transducer and activator of transcription 2 (STAT2) and ultimately promotes the ubiquitination of the signal transducer and activator of transcription 1 (STAT1) (Precious et al., 2005), thereby negatively regulating the IFN signaling pathway. Notably, a conserved Trp motif (W-X3-W-X9-W) lies upstream of the zinc finger domain, and it is also involved in the degradation of the STAT1 and inhibition of the IFN signaling pathway (Nishio et al., 2005). In addition, some studies have revealed that the CTD of the V protein is an oligomerization domain that can mediate the oligomerization of homologous or heterologous V proteins, forming V protein-dependent degradation complexes (VDC) and then rapidly degrading the STAT1 (Ulane et al., 2005).

The NTD of the V protein is not specific; it is similar to that of the P protein, which did not initially attract attention from scientists. However, with the deepening of research into the V protein, scientists have found that its NTD plays an indispensable role in its function. Studies have revealed that there are several key amino acids in the NTD of the V protein that are involved in the binding of the V protein to RNA (K74, K76, K77, R79, K81) (Lin et al., 1997), regulation of viral RNA transcription (L16, I17) (Yang et al., 2015), degradation of STAT1 (Y26, L50, L102) (Chatziandreou et al., 2002) (Table 1).

**Table 1:** Contributions of key amino acid residues in NTD to the functions of V protein.

Key amino acid residues in NTD	Functions	References
K74, K76, K77, R79, K81	Assisting V protein bind to RNA	Lin et al. (1997)
L16, I17	Regulating viral RNA transcription	Yang et al. (2015)
Y26, L50, L102	Participating in the degradation of STAT1	Chatziandreou et al. (2002)

## 4 Current progress on the regulation of viral growth and replication by the V protein

### 4.1 The role of the V protein in the viral life cycle

Studies have shown that in CPIV-infected cells, the V protein is distributed in both the cytoplasm and nucleus (Precious et al., 1995), indicating that the V protein may be a multifunctional viral protein and participate in multiple processes of the viral life cycle. Lin et al. used the SV5 minigenome system to show that the V protein inhibited CPIV-RNA replication and transcription (Lin et al., 2005). Sun et al. found that the V protein interacted with the Akt kinase in host cells and promoted virus replication by phosphorylating the P protein (Sun et al., 2008). In addition, the recombinant SV5 virus in which the V protein does not harbor a CTD replicated slowly in infection-susceptible cells and became a pseudovirus after multiple passages, indicating the importance of the V protein for CPIV replication (He et al., 2002).

### 4.2 Interactions between the V protein and other CPIV viral proteins

Generally, the structural and nonstructural proteins of a virus cooperate to facilitate viral replication and spread throughout the viral life cycle. Studies have shown that the V protein interacts with the NP protein to ensure the solubility of the NP protein before it binds to the viral genome, but the P protein inhibits the binding of the V protein to the NP protein, indicating that the binding site may lie in the NTD shared with the P protein (Precious et al., 1995). It was also revealed that the mutation of three amino acids in the N-terminus of the V protein led to the aggregation of the NP protein and formation of inclusion bodies and reduced the expression of the HN protein (Chatziandreou et al., 2002). In addition, the NTD of the V protein regulates viral RNA transcription by regulating the NP-P interaction, and amino acid residue V120 in the V protein is a key site in this interaction (Yang et al., 2015). In addition, the V protein binds to the L protein and contributes to viral genome replication regulation (Nishio et al., 2008).

## 5 Research progress into the interaction between the V protein and a host

### 5.1 The V protein promotes viral replication and growth by delaying host cell division, inhibiting cell apoptosis, promoting actin synthesis, and antagonizing tetherin

**Figure 2:**
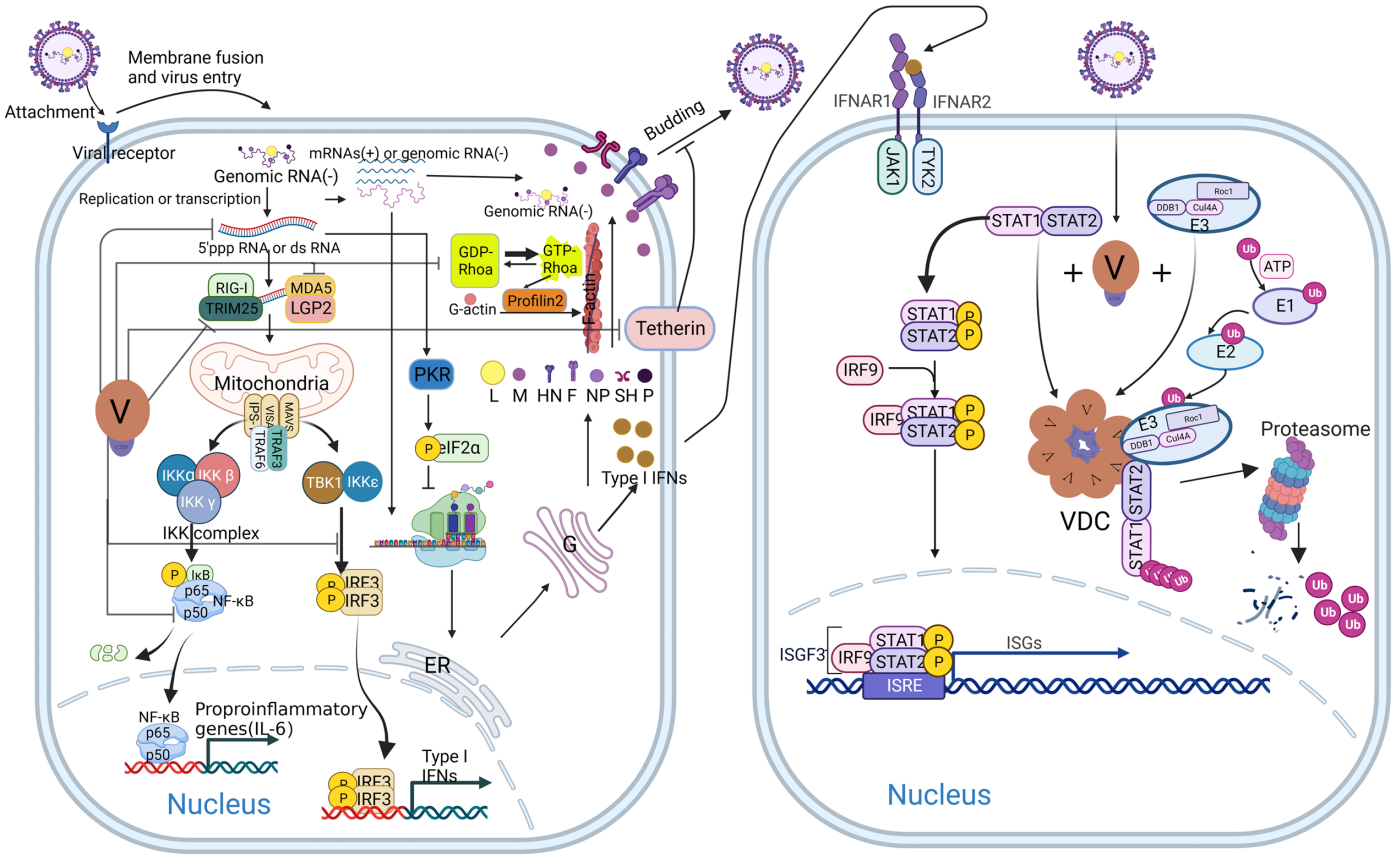
Host antiviral response and multiple antagonistic effects of V protein after CPIV infection. V protein inhibits type I IFN (IFN I) production by antagonizing TRIM25, MDA5, LGP2, TBK1/IKKε, and NFκB and promotes viral replication, budding, and spread by reducing the production of dsRNA, inhibiting tetherin, and binding Rhoa (left). V protein inhibits the IFN response by forming VDC to degrade STAT1 (right).

After infecting cells, viruses generally hijack host cells and drive preferential replication of the virus. In CPIV-infected cells, the V protein delays the cell cycle by interfering with the phosphorylation and dephosphorylation of retinoblastoma protein (RB), promotes the transport of virus envelope glycoproteins HN and F to the cell membrane, and then promotes the assembly and budding of the virus (Lin and Lamb, 2000). In addition, the V protein elevates the induction of endoplasmic reticulum (ER) stress and inhibits the activation of cysteine aspartate-specific protease 12 (caspase 12) activity, thereby blocking cell apoptosis (Sun et al., 2004). Wansley et al. found that the recombinant SV5 virus with a mutation in the amino terminus of the P/V gene (rSV5-P/V-CPI^−^) caused the apoptosis of infected cells and activated the host IFN signaling pathway. The V protein in a coinfected wild-type virus (rSV5-WT) functions as a trans-acting factor to block the apoptosis and IFN signaling pathways induced by rSV5-P/V-CPI^−^ (Wansley et al., 2003). In addition to affecting the cell cycle and inhibiting apoptosis, the V protein regulates the actin-binding protein Profilin2 by binding to Rhoa (Ras homolog gene family member A) and promotes the elongation of F-actin, thereby promoting the growth and replication of the virus (Figure 2 left) (Ohta et al., 2020).

Tetherin, also known as bone marrow stromal cell antigen 2 (BST2) or CD317, is a Type II transmembrane glycoprotein (Berry et al., 2018). Studies have shown that Tetherin is an antiviral factor that is activated and anchored to the cell membrane by type I interferon (IFN-I) and type II interferon (IFN-II) (Foster et al., 2017). When the virus buds, the glycol phosphatidyl-inositol (GPI) anchor at the C-terminus of Tetherin binds to the virus, anchoring it to the cell membrane and initiating endocytosis, promoting the transport of the virus to lysosomes for degradation. Then, pattern recognition receptors (PRRs) recognize the virus in the inner compartment and activate downstream signaling pathways to induce IFN-I responses (Zhao et al., 2022). The V protein of CPIV binds to and antagonizes tetherin, thereby promoting viral budding and spread (Figure 2 left) (Ohta et al., 2017).

### 5.2 V protein maintains efficient viral protein synthesis by inhibiting PKR activation

During viral infection, most host cells recognize and bind the double-stranded RNA produced during viral replication through protein kinase R (PKR), which is then activated through homodimerization and autophosphorylation. Ultimately, it phosphorylates eIF-2α and inhibits protein translation (Chukwurah et al., 2021). For CPIV, the V protein limits the activation of PKR by reducing the production of aberrant viral mRNA and double-stranded viral RNA (Figure 2 left), thereby maintaining the protein translation process of the virus and host cells (Gainey et al., 2008a).

### 5.3 Interactions between the V protein and host immune system

#### 5.3.1 The V protein facilitates CPIV escape from the host innate immune response

The host innate immune response refers to the first line of defense against the invasion of pathogenic microorganisms in hosts, including humans and animals. They mainly recognize the pathogen-associated molecular patterns (PAMPs) of invading pathogens through PRRs, produce IFNs and a series of cytokines to resist invasive viruses, and ultimately initiate and participate in the adaptive immune response (Kikkert, 2020). Many studies have shown that the CPIV-V protein inhibits the host innate immune response mainly by inhibiting the production of IFN and blocking the IFN antiviral signaling pathway (Figure 2) (He et al., 2002; Young and Parks, 2003; Sun et al., 2004; Precious et al., 2005; Douglas et al., 2021).

After viral infection, the PRRs melanoma differentiation-associated gene 5 (MDA5), retinoic acid-inducible gene-1 (RIG-1) and laboratory of genetics and physiology 2 (LGP2) are activated in cells and identify viral dsRNA or double 5′ capped-pppRNA structures (Negishi et al., 2018). They then activate the downstream virus-induced signaling adaptor (VISA)/mitochondrial antiviral signaling protein (MAVS)/interferon β (IFN-β) promoter stimulator 1 (IPS-1) complex, which mediates two distinct signaling pathways to defend against viral infection (Figure 2 left) (Vignuzzi and López, 2019; Iuliano et al., 2021; Zheng et al., 2023). One pathway recruits the IKK-related TANK-binding kinase 1 (TBK1) by interacting with tumor necrosis factor receptor-associated factor 3 (TRAF3) and inhibitor of nuclear factor κB kinase-ε (IKKε); these factors form a complex on the mitochondria, which activates the IFN regulatory factor 3 (IRF3) by phosphorylating it. The second pathway recruits the tumor necrosis factor receptor-associated factor 6 (TRAF6) and nuclear factor-κb (NF-κB) inhibitor IKK complex to phosphorylate the inhibitor of κB (IκB), after which phosphorylated IκB is recognized by the β-Transducin repeats-containing protein (β-TrCP), the substrate recognition subunit of the SCF (SKP1, CUL1, and F-box protein)-type E3 ubiquitin ligase complex and is then cleaved by the proteasome to release NF-κB. Finally, activated IRF3 and NF-κB are transported to the nucleus to promote the expression of IFNs and proinflammatory cytokines (Hou, 2021; Ke, 2023). After secretion, IFN binds to specific receptors on the cell surface to activate the intracellular Janus kinase/signal transducer and activator of transcription (JAK/STAT) signaling pathway, which ultimately leads to the expression of hundreds of interferon-stimulated genes (ISGs), activating host immune responses and inflammation (Figure 2 right) (Ke, 2023).

However, in CPIV-infected cells, V proteins can inhibit upstream signaling pathways in several ways to antagonize the host antiviral response and promote viral replication. First, the CTD of the V protein can bind to the minimal V protein binding region (MVBR) of MDA5 and inhibit its ATP hydrolysis activity, thereby inhibiting the normal folding and aggregation of MDA5 to form a filamentous structure, which makes it unable to activate the downstream VISA/MAVS/IPS-1 complex (Motz et al., 2013; Mandhana et al., 2018). In addition, the V protein interacts with LGP2 in the same manner, inhibiting the potentiating effect of LGP2 on MDA5 signaling (Rodriguez and Horvath, 2014). Second, the V protein can be phosphorylated as an alternative substrate for IKKε/TBK1, thereby inhibiting IKKε/TBK1 activation of IRF3 (Lu et al., 2008). These two effects eventually lead to a decrease in the synthesis of IFN-I. Notably, an article published in 2018 reported that the V protein interacts with RIG-1 and its regulatory protein tripartite motif-containing 25 (TRIM25) and inhibits RIG-1 downstream signaling pathways by inhibiting TRIM25 ubiquitination of RIG-1 (Sánchez-Aparicio et al., 2018). However, previous studies have suggested that the V protein does not interact with RIG-1 (Childs et al., 2007); these differences may have been due to the different research methods used in the two studies. The interaction between the V protein and RIG-1 needs to be confirmed by further studies. In addition, the V protein interacts with the DDB1 (Lin et al., 1998) and STAT2 (Parisien et al., 2002; Omagari et al., 2021) and then forms a globular VDC through its C-terminal oligomerization domain; DDB1 then interacts with Rbx1 (a small RING finger protein) and Cullin 4a (Cul4a) in the E3 ubiquitin ligase complex to ubiquitinate the STAT2 and ultimately inhibit JAK–STAT signaling (Ulane et al., 2005). Notably, the conserved cysteine-rich domain in the C-terminus of the V protein plays a decisive role in these processes. The loss of this domain leads to a large amount of IFN-β production and recovery of the IFN signaling pathway, resulting in extensive cytopathic effects (CPEs) (He et al., 2002).

In addition to inhibiting the synthesis of IFN-β and blocking the IFN signaling pathway, the V protein also inhibits the activation of the NF-κB family p50 protein in a virus-specific manner (Figure 2 left), thereby inhibiting its transcriptional activation of interleukin-6 (IL-6) (Lin et al., 2007). In contrast to HPIV2, the CPIV-V protein inhibits the secretion of the proinflammatory cytokines interleukin-8 (IL-8) and macrophage chemoattractant protein-1 (MCP-1) (Young and Parks, 2003) in an NF-κB-independent manner. However, CPIV-V exerted no significant effect on the secretion of the CC chemokine RANTES (Young et al., 2006). The V protein is involved in regulating the secretion of proinflammatory cytokines, chemokines and other cytokines.

Recent studies have shown that virus-mediated autophagy activation negatively regulates the host innate immune response mediated by PRRs such as RIG-1 and MDA5, thereby promoting virus replication (Wu et al., 2021; Ke, 2023). In addition, in a study of Sendai virus (SeV), autophagy-related receptor coiled-coil domain containing protein 50 (CCDC50) inhibited the recruitment of downstream MAVS proteins by RIG-1/MDA5 and targeted them for autophagic degradation. Moreover, CCDC50 inhibited the IFR3/7-mediated IFN response and NF-KB-mediated inflammatory response (Hou et al., 2021). It can be concluded from these studies that autophagy plays an important role in the virus-induced host innate immune response, similar to the role of V protein. However, there is no report on the interaction between the V protein and autophagy, and further exploration and research are needed.

#### 5.3.2 The V protein assists CPIV in escaping the host adaptive immune response

Dendritic cells (DCs) are key hub cells connecting host innate immunity and adaptive immunity. Immature DCs sense a virus through PRRs, including RIG-1 and MDA5, and then initiate the innate immune response, which promotes the expression of IFN-I, costimulatory molecules CD80 and CD86 and cytokines on the surface of DC cells and contributes to DC maturation. Mature DCs then migrate to lymph nodes, promote the maturation of naive T cells, and eventually activate the host adaptive immune response (Jr and Dl, 2021). Previous research results showed that recombinant CPIV (rCPIV) generated by V gene mutation promoted DC maturation, but wild-type CPIV did not. Only high-MOI (50 pfu/cell) rCPIV-infected DCs underwent DC maturation (CD80, CD86 expression), and infection at a low MOI (10 pfu/cell) caused only high expression of CD86 and partial maturation of DCs (Pejawar et al., 2005; Parks and Alexander-Miller, 2013). Therefore, the V protein plays an important role in promoting the maturation of DCs and further inhibiting host adaptive immunity.

In addition, CPIV (CPI^−^) generated by three amino acid mutations in the N-terminus of the V protein was less susceptible to killing by killer T cells or antibody-mediated killing than wild-type virus (CPI^+^), which allowed CPI^−^ to establish long-lasting infection in a host. This study proposed a model of virus persistence: during the interaction between CPIV and the host, the virus selectively evolved into a CPI^+^/CPI^−^ virus via V gene mutation on the basis of the host adaptive immunity status to establish persistent infection (Chatziandreou et al., 2002). The V gene clearly plays an important role in virus evolution and persistent infection.

#### 5.3.3 Could V protein antagonize the production of viral DVG?

Virus-defective genomes (DVGs) are products of the viral genome caused by mutation, modification and truncation during replication. In the absence of standard virus coinfection, DVGS cannot replicate normally in cells (Vignuzzi and López, 2019). There are two main forms of DVGs in RNA viruses: deletion DVGs and snapback or copyback DVGs (Manzoni and López, 2018). The former refers to a mutation in which the middle sequence of a virus is deleted but the two ends of the viral genome are retained, and the latter refers to a mutation in which the viral genome carries a viral 5′ structure and a loop. Snapback or copy-back DVGs are more common in RNA viruses than in DNA viruses (Genoyer and López, 2019). In infected cells, DVGs promote the establishment of persistent viral infection, induce the production of defective virions, and activate the host innate immune response (Manzoni and López, 2018; Ziegler and Botten, 2020). In paramyxoviruses, the C protein of MeV, Sendai virus (SeV) and human parainfluenza virus type 1 (HPIV1) limit the production of DVGs, but the accumulation of DVGs in cells can inhibit the expression of C protein (Genoyer and López, 2019), suggesting that there may be multiple modes of mutual regulation between paramyxovirus C protein and viral DVGs. As mentioned above, the V protein of CPIV inhibits host innate immune responses through multiple pathways; however, consistent with the paramyxovirus described above, CPIV generates snapback DVGs during replication and induces host innate immune responses during replication (Wignall-Fleming et al., 2020). However, similar to the C protein, the V protein is a product of RNA editing of the P gene (Siering et al., 2022). Whether the V protein inhibits the production of viral DVGs is unclear, and this possibility and the intrinsic mechanism need to be further studied.

## 6 V protein application to tumor immunotherapy

### 6.1 Application of P/V-CPI- to oncolytic virotherapy

Oncolytic viruses (OVs) are new types of tumor immunotherapies that replicate and lyse tumor cells through different regulatory mechanisms without affecting normal cell growth (Lin et al., 2023). Most oncolytic viruses being studied (adenoviruses, poxviruses, herpesviruses, reovirus and coxsackieviruses) are genetically edited to increase their tropism and reduce their virulence in non-tumorigenic host cells (Raja et al., 2018; Huang et al., 2023; Jiang et al., 2023). In recent years, oncolytic viruses among paramyxoviruses have been extensively studied. Through attachment protein on the surface of the virus, a virus specifically infects and kills tumor cells. For example, the recombinant measles virus that specifically binds to Nectin4 but not to the original receptor SLAM (rMV-SLAMblind). rMV-SLAMblind specifically infects and lyses tumor cells that express high levels of Nectin4 (Matveeva and Shabalina, 2020; Moritoh et al., 2023).

#### 6.1.1 Advantages of P/V-CPI- as an OV

Many studies have focused on the application of CPIV to oncolytic therapy. Initially, Wansley and Parks discovered that naturally occurring mutations in the P/V overlapping gene region could convert CPIV into a cytopathic virus (P/V-CPI-), which induced IFN production and apoptosis (Wansley and Parks, 2002). Thus, the oncolytic CPIV virus used in subsequent studies was P/V-CPI-. P/V-CPI- also induced proinflammatory cytokines and caused severe CPE in infected cells, which eventually underwent apoptosis (Wansley and Parks, 2002; Dillon et al., 2006). Currently, numerous studies have shown that P/V-CPI- increases the killing effect on tumor cells by promoting the production of double-stranded RNA, activating caspase-dependent death pathways, interfering with the DNA damage repair response in tumor cells, abrogating PKR-mediated protein synthesis, and forming extensive syncytia, etc., (Wansley et al., 2003, 2005; Dillon et al., 2006; Gainey et al., 2008a; Kedarinath and Parks, 2022).

A concerning danger of oncolytic viruses for cancer therapy is the specificity of the virus in targeting tumor cells. Surprisingly, P/V-CPI- could induce apoptosis in most human tumor cell lines currently studied in the laboratory but did not affect the growth of normal cells (Wansley et al., 2005). Meanwhile, P/V-CPI-has two major advantages as an OV. First, P/V-CPI- could induce the production of IFNs but maintain normal IFN signaling pathways, suggesting that IFN responses within normal cells limited viral growth and spread on normal cells rather than tumor cells, in which case IFN responses are dysfunctional (Capraro et al., 2008). Second is an advantage of the genome itself: CPIV is a cytoplasmic-replicating negative sense RNA virus, whose life cycles lack a DNA stage, thus posing a minimal risk of recombining the host genome (Wang et al., 2023). Moreover, there is no pathogenic gene in the CPIV genome, making it safe for infection and treatment. Correspondingly, *in vivo* experiments showed that the virus-infected mice did not have obvious pathological characteristics and could significantly reduce the tumor burden of mice (Capraro et al., 2008; Gainey et al., 2008b), indicating that the recombinant virus has high safety for mammals and has potential for clinical transformation.

#### 6.1.2 Preclinical study of P/V-CPI- as an OV

Researchers from Griffith D. Park’s team have investigated the viability and mechanisms of rCPIV as an oncolytic virus (Table 2). Preliminary studies found that P/V-CPI- was oncolytic and reduced tumor burden in mice. Further research disclosed that P/V-CPI- activated the caspase apoptosis pathway and formed syncytia to kill tumors; surprisingly, it is safe for normal or benign human cells (Capraro et al., 2008; Gainey et al., 2008b; Kedarinath and Parks, 2022). To improve its killing ability, a mutation (G3A) was introduced at the third position of the F protein fusion peptide, resulting in increased fusogenicity and tumor killing through necrosis and inflammatory responses. Notably, G3A mutation did not affect viral susceptibility to IFNs. However, F protein-induced cell death may lead to a strong immune response, which could affect the growth and dissemination of lysogenic viruses (Gainey et al., 2008b).

**Table 2:** The application of P/V-CPI- in the treatment of different tumors.

Tumor types	Combined therapeutic agents	Molecular mechanisms of killing tumors	References
Human lung cancer	Membrane-anchored Fc chimera (NA-Fc) and adoptive PM21-NK cells	Increasing release of TNF-α and IFN-γ cytokines from NK cells and enhancing CD16-Fc interactions, thus increasing PM21-NK cell killing ability	Varudkar et al. (2023)
	Adoptive PM21-NK cells	1. Infected cells: NK cells receptors recognize viral glycoprotein to activate NK cells 2. Uninfected cells: Those tumor cells IFN-I and IFN-III receptors bind IFN secreted from infected cells to activate NK cells	Varudkar et al. (2021)
	Histone deacetylase inhibitors (scriptaid)	1. Increasing in cell killing through increasing in caspase-9 and − 3/7 activity 2. Enhancing spread of the P/V-CPI- by reducing nuclear localization of IRF-3 and then downregulating IFN-β induction	Fox and Parks (2019)
Human prostate cancer	Hyperfusogenic F Protein	Increasing cell-killing by enhancing cancer cell–cell fusion	Gainey et al. (2008b)
Neuroblastoma	DNA methyltransferase inhibitor (5-azacytidine)	Enhanced cancer killing by inducing dsRNA production and activating downstream signaling (hypothesis)	Kedarinath et al. (2023)
Human laryngeal cancer	DNA-damaging agents (cisplatin)	Inhibiting DDB1 protein entry into the nucleus, leading to DNA damage-induced death	Fox and Parks (2018)

DNA-damaging drugs, such as cisplatin, are general chemotherapeutic drugs for tumor treatment. However, some tumors develop strong drug resistance during clinical treatment, which is mostly related to the gradual enhancement of the DNA repair ability in tumor cells, leading to tumor recurrence in patients (Agarwal and Kaye, 2003; Eskander et al., 2023). It is crucial to address this drug resistance to improve the efficacy of chemotherapy. Therapies combining P/V-CPI- with DNA-damaging drugs showed that virus infection inhibited DDB1 nuclear translocation, altered DNA repair pathways, and increased apoptosis, thus inducing the death of tumor cells (Fox and Parks, 2018). This finding is a promising new strategy for treating drug-resistant tumors. The combination of P/V-CPI- with histone deacetylase (HDAC) inhibitors and DNA methyltransferase inhibitors (DNMTi) also improved its killing efficacy and expanded its applicability (Fox and Parks, 2019; Kedarinath et al., 2023).

It has been established that utilizing 3D spheroid cultured tumor cells is a more effective research model for investigating cancer therapies when compared to conventional 2D cultured cells (Jensen and Teng, 2020). In one study using 3D spheroid lung cancer cells, researchers discovered that combining P/V-CPI- with PM21-NK adoptive therapies effectively killed cells of 3D spheroid tumors through activated NK cells. Notably, NK cells killed cells within spheroids that were not infected by oncolytic viruses. This was achieved through IFN-I/IFN-III released by surface cells infected with P/V-CPI- (Varudkar et al., 2021). Additionally, delivering a novel membrane-anchored Fc chimera (NA-Fc) to P/V-CPI-infected tumor cells enhanced the killing effect of adoptive NK cell killing through antibody-dependent cellular cytotoxicity (ADCC) (Varudkar et al., 2023). In conclusion, the oncolytic effect and killing mechanism of P/V-CPI- have been investigated in various tumor cells, such as lung and prostate cancers. Nevertheless, additional experiments must be carried out to verify the safety and half-life period of the treatment for future clinical studies.

#### 6.1.3 Existing problems and prospects of P/V-CPI- as an OV

As studies on CPIV have progressed, researchers have identified potential risks and problems with P/V-CPI- as an OV. While acute infection can cause apoptosis in tumor cells, some cells survive and transition to persistent infection (PI) cells over time (Gainey et al., 2008b). This poses a risk of tumor escape and recurrence. Additionally, PI tumor cells become resistant to the killing effects of adoptive NK cells, reducing the efficacy of adoptive NK cell therapy (Varudkar et al., 2023). To address these risks, it is suggested to combine chemotherapeutic drugs targeting DNA damage or delivery of NA-Fc to P/V-CPI-infected tumor cells with oncolytic viruses to enhance their efficiency (Fox and Parks, 2018; Varudkar et al., 2023). Further research is needed to understand the mechanisms by which viruses establish PI, monitor the occurrence of PI cells in real time, and optimize viral vectors to reduce the likelihood of PI cells appearing. Adoptive cell transfer (ACT) therapies are procedures that transfer qualified and active antitumor lymphocytes (T/NK cells), which are cultured *in vitro*, to patients for tumor regression. These therapies include chimeric antigen receptor T/NK cell (CAR-T/NK) therapy, T-cell receptor-engineered T-cell (TCR-T) therapy, and tumor-infiltrating lymphocyte (TIL) therapy (Lin et al., 2023). Saul J. Priceman discovered that recombinant oncolytic poxviruses containing truncated CD19 (CD19t), a tumor-specific antigen, can promote the labeling of tumor cells by viral infection, leading to improved targeting of CAR-T cells. Furthermore, the clearance of targeted tumor cells by CAR-T cells contributes to virus release, leading to the establishment of PI in these cells, which increases the duration of OV (Park et al., 2020). It is evident that the viral vector composition can be optimized, leading to lysis and death of PI tumor cells. Meanwhile, in combination with ACT therapies, PI can also become a good weapon for tumor killing.

Safety is an important consideration for all new therapies. As previously discussed, P/V-CPI- was first employed as an OV because viral growth and spread in normal cells were considerably hindered by the IFN response. It is important to test the integrity of IFN synthesis and downstream signaling pathways in tumor cells before deploying P/V-CPI- for tumor therapy. Gainey et al. improved the oncolytic effect of P/V-CPI- by introducing a hyperfusogenic F Protein to P/V-CPI- through G3A mutation. Additionally, her findings show that the mutation did not affect viral IFN sensitivity (Gainey et al., 2008b). This also implies that, during the optimization of OV vectors, it is important to test their IFN sensitivity. Even though P/V-CPI- does not have a oncolytic effect on normal or benign human cells, however, due to its extensive host tropism, highly mutable RNA viral genome, and capacity for recombination with wild-type strains make it necessary to implement several safety measures for its enhancement. These measures encompass strict strain screening, virus mutant detection, and genetic recombination detection, etc.

In conclusion, while numerous studies have shown the potential of P/V-CPI as an OV, they have only been conducted *in vitro* at the cell level. Therefore, more comprehensive research is needed to determine the oncolytic impact of P/V-CPI on various types of tumors *in vivo* and the immune response changes initiated by P/V-CPI, as well as the organism’s long-term well-being. In addition, oncolytic paramyxoviruses (Measles virus, Newcastle disease virus) currently being tested in clinical trials have implemented multiple strategies to enhance their efficacy and safety, such as fusing single-chain antibodies targeting tumor-specific antigens to enhance viral targeting (Yaiw et al., 2011), inserting exogenous cytokine genes to trigger immunogenic cell death (ICD) in tumor cells (Huang et al., 2020), and constructing viral vectors expressing bispecific T-cell engagers (BiTE) or trispecific T cells (TriTE) that activate T-cell immunity directly, which bypass the antigen-presenting cells (APCs) antigen presentation process and improve the killing ability (Freedman et al., 2017; Lin et al., 2023), and combination therapy with immune checkpoint inhibitors (ICIs) (Zamarin et al., 2014). Together, continuously optimizing the composition of P/V-CPI viral vectors, along with attempting to combine them with different therapies, will enhance the targeting selectivity, application prospects, and oncolytic ability of P/V-CPI-. Meanwhile, experiments are needed to confirm its safe dosage and duration for the next step of possible clinical studies.

### 6.2 Application of the V protein in the self-amplifying RNA vaccine

**Figure 3:**
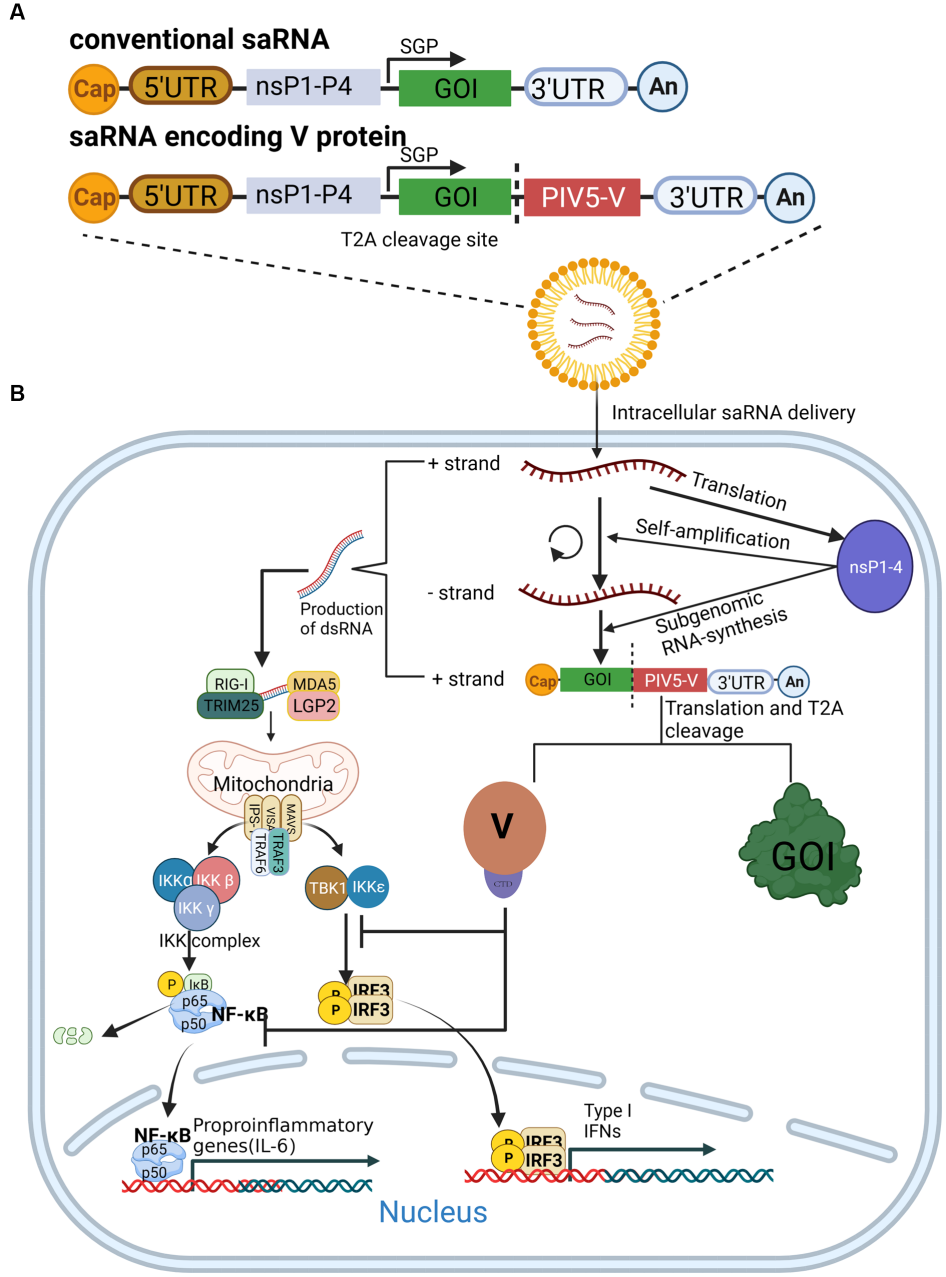
The expression of saRNA is enhanced by the V protein. **(A)** Composition of conventional saRNAs and V-protein-encoding saRNAs. **(B)** V protein antagonizes the innate immune response by inhibiting NF-κB and IFR3 activation, which in turn enhances saRNA expression.

The development of messenger RNA (mRNA) vaccines, or mRNA technology, has advanced dramatically with the advent of the coronavirus disease 2019 (COVID-19) pandemic. Notably, Karikó et al. were awarded this year’s Nobel Prize in Physiology or Medicine for their discoveries concerning nucleoside base modifications (Karikó et al., 2005), which made possible the development of an effective mRNA vaccine against COVID-19. The self-amplifying RNA (saRNA) vaccine, an emerging mRNA vaccine derived from the genome of α virus that comprises an mRNA sequence of α virus replicase and antigen protein, is able to self-amplify the gene of interest (GOI) sequence *in vivo* (Figure 3A) (Schmidt and Schnierle, 2023). In recent years, saRNA vaccines have received widespread attention in the field of infectious diseases and tumor treatment (Ballesteros-Briones et al., 2020; Lundstrom, 2020; Karam and Daoud, 2022)^.^ The main advantage of saRNA is that a lower dose of vaccine can obtain the same protective efficiency as a conventional mRNA vaccine(Papukashvili et al., 2022). With further study of saRNA vaccines, it has been shown that saRNA induces side effects. Since saRNA replicates itself *in vivo*, it produces a large amount of RNA, particularly double-stranded RNA in the cytoplasm, which is recognized by multiple PRRs, including RIG-I, MDA5, Toll-like receptor3 (TLR3), TLR7, PKR, and 2′-5′oligoadenylate synthetase (OAS), and then induces remarkable IFN responses and pro-inflammatory cytokine release (Figure 3B), which inhibits the translation of saRNA (Linares-Fernández et al., 2020; Comes et al., 2023)^.^

To date, strategies to resolve these issues have been described in detail, the most promising of which is to introduce the mRNA sequences of innate inhibiting proteins (IIPs) to saRNAs, which enable their escape from host immune recognition and the IFN response(Devasthanam, 2014). Studies have revealed that the V protein of CPIV promotes the expression of a saRNA protein by inhibiting the activation of NF-κB and IRF3 (Figure 3B), thereby increasing the expression of saRNA vaccines (Blakney et al., 2021). However, there is no evidence indicating that the V protein could enhance the immunogenicity of tested animals. Encouragingly, V protein was found to increase the proportion of saRNA-expressing cells in human skin explants(Blakney et al., 2021), suggesting that it may enhance the immunogenicity of saRNA in human cells, but more experiments are needed to confirm this.

Overall, from the present point of view, the introduction of V protein into the saRNA vector backbone indeed helps to suppress the level of innate immunity *in vivo*, which in turn enhances the expression level of the target antigen. However, because RNA viral genomes are highly mutated and prone to recombination, more experiments should be designed to verify their safety, especially their sensitivity to different types of human cells, before clinical application.

## 7 Interaction of the V protein with other viruses

**Figure 4:**
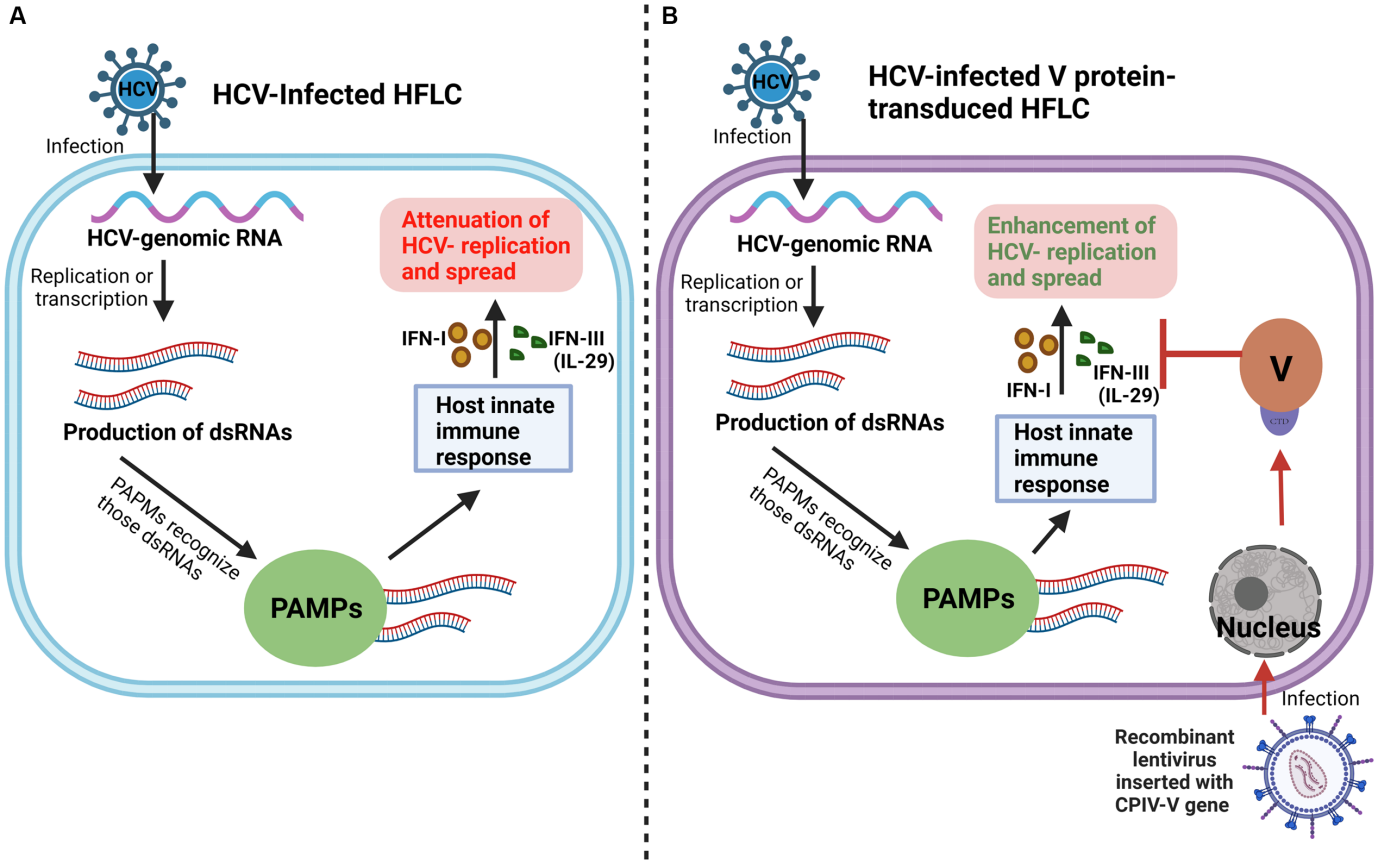
V protein promotes HCV replication and spread. **(A)** Conventionally, in HCV-Infected HFLC, PAMPs recognize dsRNAs derived from HCV replication, activate the HFLC innate immune response, and inhibit HCV replication and spread. **(B)** When the V protein is introduced into HFLC by a lentiviral vector, the V protein promotes the replication and spread of HCV by antagonizing IFN-I and IFN-III (IL-29) induced by the HFLC innate immune response.

As mentioned above, the V protein can promote the replication and spread of CPIV by inhibiting apoptosis, hijacking the host PKB kinase, and interfering with the host immune response and other processes. Some studies have shown that in primary human fetal liver cells (HFLC), the V protein promotes the expression of the hepatitis C virus (HCV) protein by inhibiting the IFN pathway and antagonizing the induction of type III IFN, IL-29, ultimately promoting the replication and spread of HCV (Figure 4) (Andrus et al., 2011). This finding indicates that the V protein is beneficial for elucidating the mechanism of other viral infections, particularly viral replication, spread and other processes.

## 8 Conclusion and future perspectives

With in-depth research on the paramyxovirus V protein, research into the CPIV-V protein has gradually become a research hotspot. Based on the above studies, it can be seen that the V protein may not only be a nonstructural protein of the virus but also function as a structural protein that participates in multiple processes of the virus life cycle and facilitates viral escape from the host immune response by delaying the cell cycle, inhibiting cell apoptosis, resisting the IFN response, inhibiting dendritic cell maturation, etc. Although the structure and function of the V protein CTD have been extensively studied, the function of the NTD of the V protein and its functional difference from the NTD of the P protein have been less intensively studied. In addition, NVGs and autophagy show a very strong correlation with the host innate immune response, and whether the V protein interacts with these components and the potential mechanism underlying this interaction and its importance need to be further studied.

Oncolytic virus and saRNA vaccines are two emerging tumor immunotherapies, and the application of the V protein shows that research into the V protein has great reference value and importance for the development of new diagnostic and treatment methods. However, V-Protein research in those fields has focused mainly on *in vitro* experiments, and many scientific experiments will be needed in the future to verify the safety, dosage and half-life *in vivo*.

## Author contributions

HCh: Data curation, Software, Writing – original draft. HZ: Investigation, Supervision, Writing – review & editing. HCa: Data curation, Methodology, Software, Writing – original draft. ML: Software, Conceptualization, Writing – review & editing. SW: Writing – review & editing, Supervision, Validation. JR: Supervision, Writing – review & editing, Investigation, Project administration.

## Glossary

CPIV: Canine parainfluenza virus
CIRD: Canine infectious respiratory disease
SV5: Simian virus 5
PIV5: Parainfluenza virus 5
NP: Nucleocapsid protein
P: Phosphoprotein
M: Matrix protein
SH: Small hydrophobic protein
HN: Hemagglutinin neuraminidase
L: Large polymerase protein
IFN: Interferon
ORF: Open read frame
HPIV2: Human parainfluenza virus type 2
NDV: Newcastle disease virus
MeV: Measles virus
NTD: N-terminal domain
CTD: C-terminal domain
DDB1: Damage-specific DNA binding protein 1
STAT2: Signal transducer and activator of transcription 2
STAT1: Signal transducer and activator of transcription 1
VDC: V protein-dependent degradation complexes
PKB: Protein kinase B
RB: Retinoblastoma protein
ER: Endoplasmic reticulum
Caspase: Cysteine aspartate-specific protease
RhoA: Ras homolog gene family member A
F-actin: Filamentous actin
BST2: Bone marrow stromal cell antigen 2
GPI: Glycol phosphatidyl-inositol
PRRs: Pattern recognition receptors
PKR: Protein kinase R
eIF-2α: Eukaryotic initiation factor 2 alpha
PAMPs: Pathogen-associated molecular patterns
MDA5: Melanoma differentiation-associated gene 5
RIG-1: Retinoic acid-inducible gene-1
LGP2: Laboratory of genetics and physiology 2
VISA: Virus-induced signaling adaptor
MAVS: Mitochondrial antiviral signaling protein
IPS-1: IFN β promoter stimulator 1
TRAF3: Tumor necrosis factor receptor-associated factor 3
IKKε: Nuclear factor κB kinase-ε
IRF3: IFN regulatory factor 3
TRAF6: Tumor necrosis factor receptor-associated factor 6
NF-κB: Nuclear factor-κB
IκB: Inhibitor of κB
β-TrCP: β-Transducin repeats-containing protein
JAK/STAT: Janus kinase/signal transducer and activator of transcription
ISGs: IFN-stimulated genes
MVBR: Minimal V protein binding region
TRIM25: Tripartite motif-containing 25
Cul4a: Cullin 4a
CPEs: Cytopathic effects
IL-6: Interleukin-6
IL-8: Interleukin-8
MCP-1: Macrophage chemoattractant protein-1
CCDC50: Coiled-coil domain containing protein 50
DCs: Dendritic cells
rCPIV: Recombinant CPIV
PI: Persistent infection
DVGs: Virus-defective genomes
OVs: Oncolytic viruses
HDAC: Histone deacetylase
DNMTi: DNA methyltransferase inhibitors
NK: Natural killer
ADCC: Antibody-dependent cellular cytotoxicity
ACT: Adoptive cell transfer
CAR-T/NK: Chimeric antigen receptor T/NK cell
TCR-T: T cell receptor-engineered T cell
TIL: Tumor-infiltrating lymphocyte
ICD: Immunogenic cell death
BiTE: Bi-specific T cell engagers
TriTE: Tri-specific T cell engagers
APCs: Antigen-presenting cells
ICIs: Immune checkpoint inhibitors
COVID-19: Coronavirus disease 2019
saRNA: Self-amplifying RNA
GOI: Gene of interest
OAS: 2′-5’oligoadenylate synthetase
TLR3: Toll-like receptor 3
TLR7: Toll-like receptor 7
IIPs: Innate inhibiting proteins
HFLC: Human fetal liver cells
HCV: Hepatitis C virus
IL-29: Interleukin-29
